# Development of a reliable questionnaire to assist in the diagnosis of fetal alcohol spectrum disorders (FASD)

**DOI:** 10.1186/1471-2431-13-33

**Published:** 2013-03-07

**Authors:** James P Fitzpatrick, Jane Latimer, Manuela Ferreira, Alexandra LC Martiniuk, Elizabeth Peadon, Maureen Carter, June Oscar, Emily Carter, Meredith Kefford, Rhonda Shandley, Harry Yungabun, Elizabeth J Elliott

**Affiliations:** 1 The George Institute for Global Health, PO Box M201 Missenden Rd, Sydney 2050, Australia; 2 Sydney Medical School, University of Sydney, Sydney, Australia; 3 The Sydney Children’s Hospital Network (Westmead), Westmead, Australia; 4 Sunnybrook Research Institute, Toronto, Canada; 5 Dalla Lana School of Public Health, University of Toronto, Toronto, Canada; 6 Nindilingarri Cultural Health Services, Fitzroy Crossing, Australia; 7 Marninwarntikura Women’s Resource Centre, Fitzroy Crossing, Australia; 8 School of Arts and Science, University of Notre Dame, Broome, Australia; 9 Indigenous Community Volunteers, Perth, Australia

**Keywords:** Fetal alcohol syndrome (FAS), Fetal alcohol spectrum disorders (FASD), Aboriginal and Torres Strait Islander, Indigenous, Questionnaire development, Diagnosis, Reliability testing, Reproducibility of results, Test-retest, Percent exact agreement

## Abstract

**Background:**

A battery of clinical assessments was used in the Lililwan* Project, Australia’s first population-based Fetal Alcohol Spectrum Disorders (FASD) prevalence study, conducted in the remote Fitzroy Valley, Western Australia. One objective was to develop and assess test-retest reliability of an acceptable questionnaire for collecting health information in remote Aboriginal communities feasible for use in the Lililwan Project.

**Methods:**

A questionnaire was developed by paediatricians to assist in diagnosis of FASD. Content was based on a literature review of FASD diagnostic criteria, existing questionnaires and risk factors for FASD and birth defects. Aboriginal community members, including qualified Aboriginal language interpreters, adapted the questionnaire to ensure language and cultural components were appropriate for use in the Fitzroy Valley. Locally developed pictorial aids were used for gathering accurate information on alcohol use. Aboriginal ‘community navigators’ assisted researchers to translate the questions into Kimberley Kriol or local Aboriginal languages depending on participant preference.

A subset of 14 questions was assessed for test-retest reliability in 30 parents/carers of children in the Lililwan Project cohort, who were interviewed by one rater using the entire questionnaire, then by a second rater who repeated 14 critical questions at least 6 hours later.

**Results:**

The full questionnaire contained 112 items and took 50 minutes to administer. For a subset of 14 items from the full questionnaire percent exact agreement between raters ranged from 59-100%, and was below 70% for only 1 question. Test-retest reliability was excellent (Kappa 0.81-1.00) for 5 items, substantial (Kappa 0.61-0.80) for 5 items, and moderate, fair or slight (Kappa ≤0.60) for the remaining 4 items tested. Test-retest reliability for questions relating to alcohol use in pregnancy was excellent. When questions had moderate, fair or slight agreement, information was obtained from alternate sources e.g. medical records. Qualitative feedback from parents/carers confirmed acceptability of the questionnaire.

**Conclusions:**

This questionnaire had acceptable test-retest reliability and could be used to collect demographic, socio-cultural and biomedical information relevant to the diagnosis of FASD in Aboriginal communities throughout Australia and elsewhere. Community input is crucial when developing and administering questionnaires for use in cross-cultural contexts.

*Lililwan is a Kimberley Kriol word meaning ‘all the little ones’. Kimberley Kriol is the main language spoken by Aboriginal people in the Fitzroy Valley.

## Background

In remote Aboriginal communities of the Fitzroy Valley historical trauma, chronic alcohol oversupply, high-risk patterns of alcohol consumption and the devastating effects of alcohol on the developing fetus threaten the continuation of language and culture (personal communication, June Oscar 2011). Alcohol is teratogenic and exposure *in utero* can cause a spectrum of lifelong physical, neurological and cognitive abnormalities termed fetal alcohol spectrum disorders (FASD), including specific diagnoses of fetal alcohol syndrome (FAS), partial FAS (pFAS) and neurodevelopmental disorder - alcohol exposed (ND/AE) [1].

In 2007, the Fitzroy Valley communities introduced local alcohol restrictions, with immediate and enduring social and health benefits [2]. Until recently few people in the Fitzroy Valley communities were aware of the effects of alcohol on the developing fetus. Since 2008 a concerted FASD awareness raising campaign has laid the foundation for FASD prevention.

This campaign is part of a sophisticated strategy to address FASD, [3] including partnering with leading research organisations The George Institute for Global Health and Sydney Medical School, The University of Sydney, to conduct Australia’s first population-based FASD prevalence study: The Lililwan Project [4-6]. This paper describes the development of a reliable questionnaire for use in the Lililwan Project.

Alcohol exposure *in utero* is the most common preventable cause of intellectual impairment, and international estimates of FAS prevalence range from a median of 0.27 cases per 1,000 people in surveillance studies, to a median of 8.5 cases per 1,000 people using active case ascertainment methods [7]. Studies in some high-risk communities that include all diagnoses on the FASD spectrum (FAS, pFAS, ND-AE) report a median prevalence of 19.0 cases per 1,000 people [7]. Communities where high-risk drinking is common, including some Fitzroy Valley communities, are expected to have high FASD prevalence rates. In Australia the prevalence of FASD is unknown and there are few FASD screening programs or diagnostic clinics [8,9]. Diagnosis of FASD requires a comprehensive history and a multidisciplinary clinical and developmental assessment [10]. A comprehensive history includes details of prenatal alcohol exposure, other pregnancy complications and exposures, birth, development, health and social/environmental conditions. Multidisciplinary clinical/developmental assessment identifies dysmorphology and growth impairment, central nervous system structure/function and differential diagnoses [10].

Prenatal alcohol exposure data is key in diagnosis of FASD. A number of tools have been developed for gathering alcohol exposure and comprehensive history data for the purpose of FASD diagnosis. FASD researchers in the Collaborative Initiative on FASD (CIFASD) have developed a standard vocabulary for alcohol exposure variables with the aim of gathering comparable data from multiple sites [11]. The University of Washington (UW) 4-digit diagnostic code [12] includes a ‘new patient information form’ to document demographics, growth, health, schooling, environmental stressors and alcohol exposure *in utero*. A series of 14 questions document alcohol exposure prior to and during pregnancy and evidence of maternal alcohol dependency.

Researchers at the University of New Mexico developed an extensive (240 item) questionnaire for use in FASD prevalence studies in rural South African communities [13]. This questionnaire includes demographic, health and antenatal items with a particular focus on family structure and household stressors including employment status, household occupancy and income, and domestic violence. Questions about maternal nutrition lead into a comprehensive series of questions about alcohol use.

While suitable for use in their intended target populations, none of the existing questionnaires are appropriate for use in remote Australian Aboriginal communities. Our questionnaire required a standardised approach to administration, tailored language with meaningful local terminology, and detailed questions on language groupings and environmental conditions including early life trauma. Additionally, specific pictorial aides were used to display local alcohol brands and improve accuracy of alcohol use reporting. This questionnaire enables history taking as part of an assessment battery to accurately establish FASD prevalence in these communities.

### Objectives

The objectives of this study were to:

1. Develop a comprehensive, culturally acceptable questionnaire feasible for use in the Lililwan project to collect demographic, socio-cultural, antenatal and biomedical data from parents/carers of children born in 2002 or 2003 and living in the Fitzroy Valley in 2010 or 2011.

2. Evaluate the test-retest reliability of this questionnaire.

## Methods

### Setting

The setting for this work is the remote Fitzroy Valley of North Western Australia, including Fitzroy Crossing town and approximately 45 remote communities representing the language groups of the Bunuba, Walmajarri/Wangkatjungka, Gooniyandi and Nyikina peoples [14]. The Fitzroy Valley is approximately 2,500 km North of Perth, and 400 km East of Broome, and includes communities within a radius of 200 km from Fitzroy Crossing town. The total population of the Fitzroy Valley is 4,500, approximately 80% being Aboriginal [14]. Kimberley Kriol is the most commonly spoken language but traditional Aboriginal languages and Standard Australian English are also used.

### Questionnaire development

A questionnaire (Additional file 1) was created to collect accurate information about pregnancy, child health and development in a cohort of predominately Aboriginal participants in the Lililwan Project FASD prevalence study. The questionnaire was initially developed in Sydney, Australia by general paediatricians with experience in FASD diagnosis and research and a paediatric advanced trainee with experience working with remote Aboriginal communities in the Fitzroy Valley. Content was informed by a literature review of FASD diagnostic criteria and existing questionnaires [12,15-19] with consideration of potential antenatal and environmental influences on child development, [20-23] maternal risk factors for birth defects, and risk factors for FASD [24-27].

The information gathered, when used in the context of a comprehensive clinical assessment enables a FASD diagnosis to be made by application of various international FASD diagnostic criteria. These include the Canadian Guidelines for the diagnosis of FASD [15], University of Washington 4-Digit Diagnostic Code, [12] Institute of Medicine FASD diagnostic criteria, [28] clarified Institute of Medicine FASD diagnostic criteria, [17] and the Centers for Disease Control Guidelines – FAS [29].

### Refining the questionnaire

From February - April 2010 researchers worked with an Aboriginal leader from Fitzroy Crossing to ensure that the questions contained in the questionnaire were culturally appropriate for use in Aboriginal communities in the Fitzroy Valley. The questionnaire was then refined in consultation with three local Aboriginal community members - ‘community navigators’ - on the research team. Input was sought from an Aboriginal representative of the Kimberley Interpreting Service (a regional Aboriginal language centre). The questionnaire was modified to take into account cultural and language considerations.

Sensitive questions about the father (which it was thought could cause female participants to feel uncomfortable) were removed. Similarly, it was advised that during interviews, questions about ‘women’s business’ (e.g. history of miscarriage, complications in pregnancy or details of mode of delivery) should not be asked in the presence of males. In order to make the interview process as non-threatening as possible, questions were ordered so that they ‘flowed’ intuitively and so that participants could anticipate the type of question that was to follow. Script was incorporated in the questionnaire to reassure participants that confidentiality would be maintained and families and children would not be identified in stored data or publications.

Language considerations included using plain English throughout, substituting local terms *‘grog’* for alcohol, *‘kid’* for child, and asking who *‘grew them up’* for who raised the child. The questionnaire was always administered in the presence of a community navigator who was able to explain questions in Kimberley Kriol or a local Aboriginal language as required and to interpret the responses.

### Scripting around sensitive questions about alcohol

A preamble was inserted prior to sensitive questions, including about alcohol use in pregnancy, and consent to continue was verbally re-established at that time:

“The next questions are about if you drank grog before and during the pregnancy with this kid, is that OK? Answering these questions might be a bit hard, but it’s really important that you’re honest about it. It’s not about shame or blame, but about helping kids who need help to be as good as they can be. If you start to feel upset we can stop.”

### Development and use of pictorial communication aids to quantify alcohol consumption

**Figure 1:**
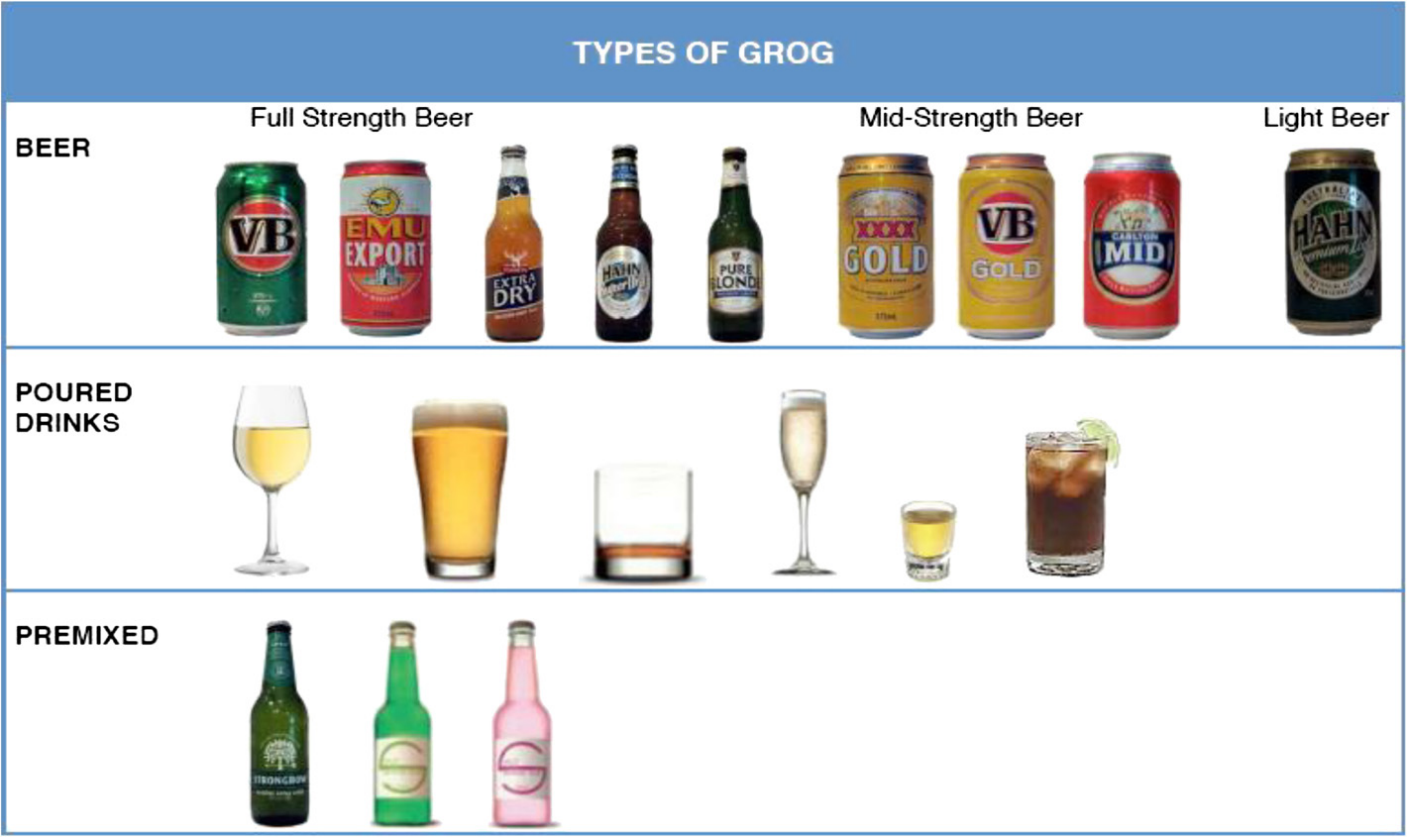
Pictorial aid for ‘type’ of alcohol consumed.

**Figure 2:**
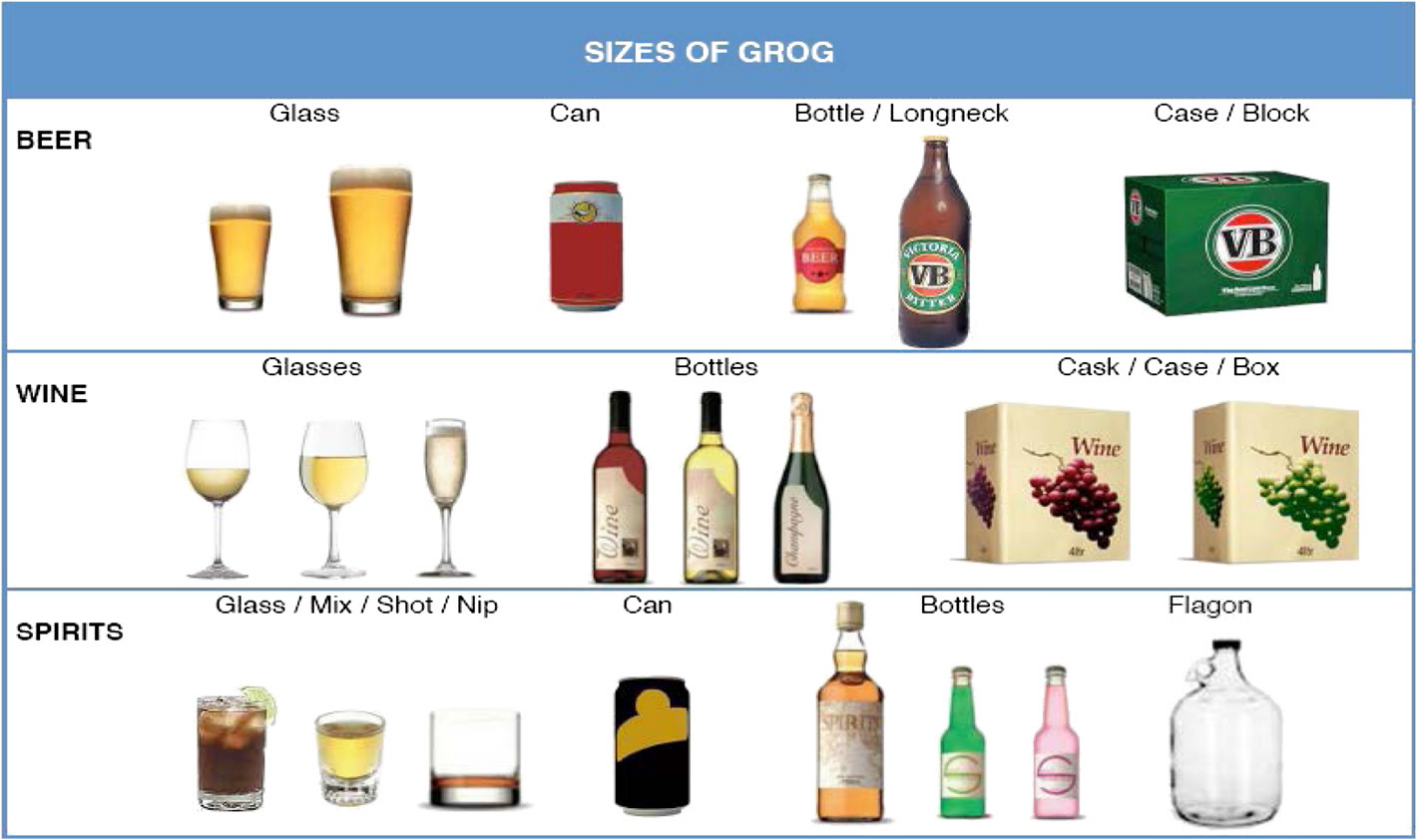
Pictorial aid for ‘size’ of alcoholic beverages consumed.

Questions about alcohol consumption specifically quantified the amount, timing and frequency of alcohol use in pregnancy, including episodes of ‘binge’ drinking. Reported intake was converted into standard drink equivalents to enable risk stratification using questions adapted from the Alcohol Use Disorders Identification Test screening system (AUDIT-C) [30]. While there are various definitions of ‘binge’ drinking, we used more than 6 standard drinks per occasion to provide a conservative estimate in communities with known high risk drinking patterns. Items relating to alcohol use in the 3 months before pregnancy and indicators of maternal alcohol dependence (e.g. alcohol-related injury or illness) were included. To improve participant recall of alcohol use in the index pregnancy 7-8 years prior, pictorial communication aids were developed using local alcohol brands so that the type and volume of alcohol consumption could be accurately described (Figures 1 and 2).

### Scripting around sensitive questions about the home environment

The questionnaire includes items relating to environmental exposures at home and early life trauma (including financial troubles, food insecurity, overcrowding and domestic violence) that may contribute to learning and behavioural impairment. A preamble was scripted prior to these sensitive questions to gain agreement to continue:

*“The next questions are about things that could have made this kid worry or feel sad while they were growing up. We know these questions might be hard for you. Is it OK to keep going?”* Questions included: *“Are there times when adults in this kid’s house worry about not having enough money (or food)?”* and *“Do adults or parents fight a lot at home?”*

### Leaving people feeling ‘safe’ and ‘good’

A concluding script prompted the rater to express their appreciation. Participants were reassured that the study would contribute to improving child health services in the community and that responses would remain confidential. Feedback was sought from participants on how the interview could be improved:

“Thank you so much for being part of this interview. Nindilingarri [Cultural Health Services], Marninwarntikura [Womens Resource Centre] and other organisations will work hard to make sure this project helps all children in the Fitzroy Valley. The information that you give us is confidential between you and the Lililwan Project workers. Were there any problems with this interview? How do you think we could do it better?”

Two raters were trained to administer the questionnaire as a structured interview in Fitzroy Crossing over a one-week period in May 2010. Raters were a paediatric advanced trainee and a volunteer with health service experience. Raters were partnered with two local Aboriginal ‘community navigators’. Raters formed two working pairs, with the non-Aboriginal rater asking questions in person, in plain English and recording verbal responses on the questionnaire, and the ‘community navigator’ interpreting as required. Interviews were not audio or video recorded.

### Pilot reliability testing

Prior to full reliability testing, inter- and intra-rater reliability was assessed in a pilot study with ten participants. Participants were a convenience sample from one remote community in the Fitzroy Valley, a sub-sample of the total Lililwan Project cohort. The same 12 questions were scored twice within the same interview in person, by two raters. Inter-rater reliability was assessed as the responses were scored by two independent raters, and intra-rater reliability was assessed as 12 questions were repeated within the same interview. The aim of this pilot was to inform the design and selection of questions for the main reliability study and to provide important information that would inform any necessary retraining of the raters.

### Full reliability testing

Between 25^th^ May and 15^th^ July 2010 a convenience sample, from 9 communities in the Fitzroy Valley, of 30 parents/carers of children in the Lililwan project cohort was recruited. Participants were predominately birth mothers (n=22), the remainder being aunts (n=5), 1 grandmother, 1 father and 1 guardian. None of this sample had participated in pilot reliability testing.

In order to assess test-retest reliability a subset of 14 questions from the original 112 item questionnaire was identified to be asked on a second occasion (questions 23, 32, 32(d), 32(e), 34, 37, 38, 39, 42, 43, 57, 58, 66, 67). (Table 1) We decided to evaluate the reliability of 14 questions (as opposed to the entire questionnaire) due to time limitations imposed by the difficulty in locating and re-interviewing participants in very remote communities. These questions were selected as they related to the pregnancy, particularly antenatal complications and alcohol exposure [30], and information about current development and educational status. Twelve of the 14 questions had been assessed in the pilot reliability study.

**Table 1:** Test-retest reliability assessment of 14 important questions (N=30 caregivers)

**Level of agreement of specific questions**	**Percent exact agreement**	**Kappa value**	**95% CI**
**Excellent agreement (Kappa 0.81-1.00)**			
Q34 Pregnancy: smoking during pregnancy n=27	100%	1.00	1.00 to 1.00
Q38 Pregnancy: alcohol use during pregnancy n=27	100%	1.00	1.00 to 1.00
Q42 Pregnancy: number of drinks on a typical day n=25	96%	0.99*	0.97 to 1.00
Q43 Pregnancy: frequency of drinking alcohol n=24	83%	0.98 *	0.96 to 1.00
Q66 Child: development behind children of same age n=30	90%	0.81	0.60 to 1.00
**Substantial agreement (Kappa 0.61-0.80)**			
Q37 Pre-pregnancy: alcohol use before pregnancy n=27	89%	0.76	0.52 to 1.00
Q32d Pregnancy: infections during pregnancy n=27	89%	0.75	0.48 to 1.00
Q67 Child: areas of developmental delay n=28	82%	0.74	0.56 to 0.92
Q39 Pregnancy: trimester alcohol consumed n=27	81%	0.73	0.54 to 0.91
Q58 Child: specifics of medical problems requiring care in question 57 n=23	87%	0.72	0.49 to 0.94
**Moderate agreement (Kappa 0.41-0.60)**			
Q32e Pregnancy: hospital admissions during pregnancy n=27	81%	0.60	0.30 to 0.89
Q57 Child: long term medical problems requiring care n=30	77%	0.55	0.31 to 0.79
**Fair agreement (Kappa 0.21-0.40)**			
Q32 Pregnancy: medical problems during pregnancy n=27	59%	0.39	0.16 to 0.63
**Slight agreement (Kappa 0.00-0.20)**			
Q23 Child: learning or behavioral support in school n=30	73%	0.03	-0.14 to 0.20

n= number of respondents to each question *For Q42 and Q43 a quadratic weighted Kappa was used.

Briefly, one rater interviewed a participant using the full 112 item questionnaire, and after a minimum of 6 hours the other rater repeated a portion of the interview using the selected subset of 14 questions. The two raters were blinded to each other’s scores. Scores from each rater were compared to determine inter-rater agreement and test-retest reliability. A minimum 6-hour period between first and repeat interview was used to minimise recall bias while balancing the practicality of locating highly mobile participants for re-interview. Full questionnaire interviews took approximately 45-60 minutes to complete, with repeat interview with 14 questions taking approximately 15 minutes. All recruited participants completed the interviews.

### Statistical analysis

In addition to percent exact agreement between the pairs of raters, Kappa values were calculated for all 14 questions, except for questions 42 and 43 which have ordered categories necessitating calculation of weighted Kappas using quadratic weighting. Quadratic weighting was used since the differences between the ‘upper’ categories were deemed to be more important than the differences between the ‘lower’ categories. [31,32]. Interpretation of the strength of agreement was based on the system proposed by Landis and Koch: [33] a Kappa value of 0.81-1.00 is excellent agreement, 0.61-0.80 indicates substantial agreement, 0.41-0.60 moderate agreement, 0.21-0.40 fair agreement, 0.00-0.20 slight agreement and less than 0.00 poor agreement. All statistical analyses were performed using MedCalc for Windows, version 12.6.0.0 (MedCalc Software, Ostend, Belgium). We calculated that 40 participants would need to be interviewed on two occasions to provide sufficient power for a Kappa value of 0.70, with 95% confidence intervals ranging from 0.50 to 0.90.

STROBE guidelines for reporting observational studies were used [34].

### Ethics approval

Ethics approval for this study was granted by the University of Sydney Human Research Ethics Committee (Approval number 12527), the Western Australian Aboriginal Health Information and Ethics Committee (Approval number 271-01/10), the Western Australian Country Health Service Board Research Ethics Committee (Approval number 2010:01), and the Kimberley Aboriginal Health Planning Forum Research Sub-committee (Approval number 2010-001). Written consent was obtained from all participants prior to participation in the study.

## Results

### Questionnaire development

The interview includes 112 questions (some with sub-questions) about child demographics, schooling, language, place of residence, living conditions, prenatal exposures (including alcohol, illicit drugs and medications), birth and neonatal history, early life trauma, health and educational outcomes and family characteristics. It also includes items to determine risk levels of alcohol consumption, birth defects, developmental problems, and syndromes with features similar to FAS. The entire questionnaire is included at Additional file 1.

### Pilot reliability testing

The average Kappa value for inter-rater reliability was 0.95 (range 0.77-1.00), indicating excellent agreement [33]. Kappa values for intra-rater reliability were consistently greater than or equal to 0.63, indicating substantial or excellent agreement in all but two questions (Q 66 and Q67). For these two questions the language used to clarify questions was modified, and raters were trained to standardise the way questions were asked and the information recorded, prior to full reliability testing.

### Full reliability testing

A total of 30 participants were recruited for the full reliability study (22 mothers, 5 aunts, 1 grandmother, 1 father, 1 guardian). Median time between first and second interview was 525 hours (mean=157 hours, range 6-1056 hours). As shown in Table 1, the agreement ranged from 59-100%, and was below 70% for question 32 (medical problems during pregnancy agreement=59%).

Kappa values for test-retest reliability ranged from 0.03 to 1.00. (Table 1) Five questions had Kappa values indicating excellent agreement (Kappa 0.81-1.00). Five questions had substantial agreement (Kappa 0.61-0.80). Four questions had moderate, fair or slight agreement including questions 32e (hospital admissions during pregnancy), 57 (child’s long term medical problems requiring care), 32 (medical problems during pregnancy) and 23 (learning or behavioral support in school). The discrepancy between the Kappa value of 0.03 and the percent exact agreement of 73% for question 23 reflects the ‘base rate problem’ relevant for a question with a high prevalence of ‘no’ responses. For example where prevalence is high the agreement needs to be close to 100% for the Kappa to reflect higher agreement [35].

### Qualitative feedback

Qualitative data about the acceptability of the questionnaire was gathered from participants by asking: “Were there any problems with this interview, and how do you think we could do it better?” 98% of respondents gave positive feedback including: “The Lililwan Project is a good thing, it’ll help with kids who have FASD and problems with learning. It is so important that kids are given a chance - it’s not their fault if they are born with problems.” In 2% of cases feedback was not positive and comments included: “The timing of interview was not good, it should have been on another day” and “I am sick of being asked questions with no help for the problems I want help with. My immediate issue is around violence – no-one is able to help.” Where carers raised issues such as violence, researchers recommended local services and made referrals with the carer’s consent if appropriate.

## Discussion and conclusions

### Questionnaire development

We have developed a comprehensive and reliable questionnaire for history taking relevant to making FASD diagnoses in Aboriginal communities. Diagnosis of FASD requires collection of accurate information relating to pregnancy exposures, birth, health and developmental outcomes in addition to a multidisciplinary clinical assessment. Our questionnaire considers language and cultural sensitivities and is acceptable to participants. It would be applicable for use in other remote Aboriginal communities in Australia and in communities elsewhere in which high risk alcohol use is prevalent.

### Pilot and full reliability testing

Pilot reliability testing confirmed substantial or excellent inter- and intra-rater agreement in 10 out of 12 questions tested. It also informed rater training in standardisation of language used and interview technique. In full reliability testing, we assessed the test-retest reliability in a subset of 14 important items from the questionnaire and found Kappa values >0.60 in ten out of 14 questions. The finding of Kappa values of ≤0.60 in the remaining 4 questions could be explained either by inconsistency in raters recording, or inconsistency of answers provided by the participant. We hypothesise that the error was more likely to arise from recall bias or participants lacking the information required to answer the questions than from rater error. For instance parents/carers may be unaware of details of support provided in the school setting and those who are not biological mothers may be unaware of problems during the mother’s pregnancy. Rater error was minimised by the use of a pilot study that provided further information for training raters ensuring a standardised interview and data recording technique. Local Aboriginal community navigators were present to interpret in local languages.

For the questions with Kappa values ≤0.60 we elected to obtain the information from an alternative source (e.g. school or hospital records) rather than by interview. This procedure was followed for the entire Lililwan FASD prevalence study cohort. For example, accurate information about learning and/or behavioural support at school was obtained from the school, rather than the parent/carer. Similarly, problems or hospital admissions during pregnancy were identified through a review of antenatal records for each mother, and details of childhood medical problems from the child’s medical records.

Notably the Kappa values indicated excellent agreement for questions relating to alcohol use in pregnancy. The exception was question 39 (trimester alcohol consumed in pregnancy, Kappa 0.73) for which substantial agreement was found. This supports our expectation that alcohol use would be reported accurately by birth mothers. Our findings are consistent with evidence from other studies indicating that retrospective reporting of alcohol consumption is more accurate than reporting at the time of pregnancy [36-39]. For instance, one study showed that the predictive validity of retrospective reporting of alcohol use (5 years after pregnancy) is high in relation to craniofacial anomalies, and higher than antenatal reporting in relation to other alcohol-related anomalies [38]. Similarly, alcohol consumption reported 14 years after a pregnancy was more predictive of behavioural problems in teenagers than reports of consumption at the time of pregnancy [37]. Taken together these findings suggest that retrospective reporting is likely to yield valid data on prenatal alcohol exposure.

To encourage participants to accurately report alcohol use during pregnancy, we took great care to emphasise confidentiality and minimise the potential for feelings of guilt or shame. One quarter of respondents were carers rather than birth mothers. We believe their responses to questions about the mother’s alcohol use in pregnancy are accurate because the carers interviewed were usually family members and lived in or near the birth mother’s household. In remote Aboriginal communities with close kinship networks and overcrowded living conditions the drinking behaviour of pregnant women is frequently observed and known by the wider community.

Other groups have reported validation or language modification of questionnaires used in FASD diagnosis. The University of Washington ‘new patient information form’ was designed for caregivers in a general United States population to self-complete. It identifies unique patterns of exposure that differentiate FAS from Alcohol Related Neurodevelopmental Disorder and correlate significantly with underlying structural and functional brain abnormalities [1,40-42]. The questionnaire used by the University of New Mexico in South African FASD prevalence studies includes ‘script’ to introduce sensitive questions and confirm consent. The questionnaire takes into account the local cultural and language context and when used is administered in Afrikaans, the primary regional language [13]. Our study is the first to report development and reliability testing of a tool for use in Australian Aboriginal communities.

Our findings are comparable or superior to test-retest reliability of the Alcohol Use Disorder Identification Test (AUDIT), a questionnaire commonly used clinically and in research relating to alcohol use. In a general population sample of 457 people in Sweden, intra-class correlation coefficients on ten individual items ranged from 0.29 to 0.80, and the Kappa value was 0.69 for agreement at a predetermined ‘risk cut-off score’ [43]. Interestingly, many tests in common clinical use show only fair inter-rater agreement. A study of specialist cardiorespiratory physical therapists in Canada showed only fair agreement (Kappa 0.26) on clinical interpretation for auscultation of breath sounds [44]. Similarly, a recent study of orthopedic surgeons about the nature of upper arm (humerus) fractures based on a variety of imaging methods, showed slight agreement on the classification of the fracture (Kappas ranged from 0.06-0.14) and fair agreement on the recommended treatment option (Kappas ranged from 0.28-0.33) [45].

A strength of our study is that the questionnaire was developed from a comprehensive literature review and modified with input from the Aboriginal community and local language interpreters to refine its language and content. Importantly, questions were included to allow application of a validated assessment tool for alcohol exposure risk (AUDIT-C) [30] and existing FASD diagnostic criteria [12,15,17]. Another strength of this study was reporting both agreement and Kappa to take into account chance agreement or disagreement between raters.

One limitation of the study is that reliability testing was only performed in a subset of questions used. This was due to logistical difficulties in locating participants for re-interview, including the remote location, lack of transport, lack of telephones in most households and high mobility between residences. We originally estimated that we would need 40 subjects to provide sufficient power for the reliability study. Our ability to recruit only 30 subjects resulted in larger but acceptable 95% confidence intervals. Recall bias may affect the accuracy of responses to questions relating to past events (up to 8 years previously in this study). This may have been the case for questions 32 and 32e that related to problems in the pregnancy.

Future research may include assessment of the validity of this questionnaire by examining associations between items in the diagnostic questionnaire and consequent FASD diagnosis. This would identify specific items with predictive validity for FASD diagnosis that could be included in a short version of the questionnaire for the purpose of screening or more targeted history taking.

This study demonstrates that a questionnaire that is based on existing literature can be modified for use in Australian Aboriginal communities with unique cultural and language characteristics. Furthermore, we report acceptable test-retest reliability of a subset of items on this questionnaire. The process followed to refine this questionnaire could be used in other populations with unique cultural and language characteristics.

## Competing interest

The authors declare that they have no competing interests.

## Authors’ contribution

JL, JPF, MF, EJE, MC, and JO consulted with the participant communities, conceived of and designed the study, and obtained ethics approval for the study. EP conducted the literature review and created the first draft of the questionnaire. EJE and JPF reviewed and created the second and further drafts of the questionnaire. MC, EC, HY and RS further refined the questionnaire for language and cultural appropriateness. MF and JL designed reliability protocols and databases for the diagnostic questionnaire. JPF, HY, RS and MK conducted participant recruitment and data collection. MF and ALCM analysed the data and contributed to interpretation of results. JPF wrote the initial drafts of the manuscript. MC, JO, EC, HY and RS are Aboriginal community members from the Fitzroy Valley. JPF, JL, MC, JO and EJE are Chief Investigators on the study. MK is a volunteer with Indigenous Community Volunteers. JO is a Master’s candidate with the University of Notre Dame, Broome, Western Australia. JPF is a PhD candidate with The University of Sydney, New South Wales. All authors read, edited and approved the final manuscript.

## Pre-publication history

The pre-publication history for this paper can be accessed here:

http://www.biomedcentral.com/1471-2431/13/33/prepub

## Supplementary Material

Additional file 1 The Lililwan Project – FASD Diagnostic Questionnaire. Click here for file
