# Supplementary material for: Development of a reliable questionnaire to assist in the diagnosis of fetal alcohol spectrum disorders (FASD)
Source: BMC Pediatr. 2013 Mar 7;13:33. doi: 10.1186/1471-2431-13-33 (PMC3680181; doi:10.1186/1471-2431-13-33)
Supplement: Additional file 1 — The Lililwan Project – FASD Diagnostic Questionnaire. [file 1471-2431-13-33-S1.pdf]

DATE ....

CHILD ID.....................

THE LILILWAN PROJECT – INTERVIEW FORM

FILL IN BEFORE THE INTERVIEW STARTS - This page will be detached and stored separately so that the identity of the child and parent/carer is kept secret.

Which community is the interview being conducted at? ____

Names of person/people collecting information (interviewer/s):

First person: ____

Second person: ____

Name of study child: ____

Age and D.O.B. of study child: ________________________________

Is child under DCP care? ☐ **Yes** ☐ **No** **If yes, DCP staff member must be at the interview.**

If yes, Name of DCP staff member: ____________________________

CONFIDENTIALITY

The information you give us is confidential between you and the Lililwan Project workers. What you say will be put only on this form without giving it to anyone else outside.

Every person doing these interviews had to sign a confidentiality form saying we are not allowed to tell anyone about what you have told us.

When we finish interviewing you, your name will be taken off and replaced with a number so nobody will know whose form it is. Your name or the child's name will not be used, only the number (ID number). The forms will be securely locked up.

Name of person / people being interviewed (interviewee/s):

What is the name of each person being interviewed, and how are each of you related to this child?

First person **First name(s)** ____

Last name(s) ____

Second person **First name(s)** ____

Last name(s) ____

Relationship to child?

☐ Stepmother	☐ Foster mother	☐ Adoptive mother	☐ Auntie	☐ Grandmother	☐ Sister
☐ Birth father	☐ Stepfather	☐ Foster father	☐ Adoptive father	☐ Grandfather	☐ Brother
☐ Uncle	☐ Cousin	☐ Carer	☐ DCP worker		

☐ Other (specify): __________________________________

Third Person **First name(s)** ____

Last name(s) ____

Relationship to child?

☐ Stepmother	☐ Foster mother	☐ Adoptive mother	☐ Auntie	☐ Grandmother	☐ Sister
☐ Birth father	☐ Stepfather	☐ Foster father	☐ Adoptive father	☐ Grandfather	☐ Brother
☐ Uncle	☐ Cousin	☐ Carer	☐ DCP worker		

☐ Other (specify): __________________________________

What language do you prefer to do the interview in? ____________________________

Is it OK to do the interview in English with the community navigator helping to explain the questions?

If interpreter required what language/day/place/time has interview been booked for? ____________________________________

DATE ....

CHILD ID......................

PAGE 2

HAS BEEN LEFT BLANK INTENTIONALLY

DATE ....

CHILD ID.....................

PART 1 – GENERAL INFORMATION ABOUT YOU AND YOUR FAMILY

1	**Mother's details**	**Mother's current age at time of interview:** ____ **years**<br>How old are you now?<br><br>**Date of birth: / /**<br>What is your date of birth?
2	**Father's details**	**Father's current age at time of interview:** ____ **years**<br>How old is the father now?<br><br>**Date of birth: / /**<br>What is his date of birth?
3	**Study ID number: Date of birth: / / Girl or Boy (circle)**	
4	Is this child of Aboriginal, Torres Strait or South Sea Islander origin?	☐ Aboriginal **Go to next Q**<br>☐ Torres Strait ☐ South Sea Islander **Go to Q 10**<br>☐ Neither ☐ Unknown **Go to Q 10**
	**IF ABORIGINAL**	
5	What's the child's (biological) father's language group?	☐ Bunuba ☐ Goonyandi ☐ Walmajarri/ Wangkatjungka<br>☐ Nykina ☐ Unknown ☐ Other (specify):
6	What's your (the child's mother's) language group?	☐ Bunuba ☐ Goonyandi ☐ Walmajarri/ Wangkatjungka<br>☐ Nykina ☐ Unknown ☐ Other (specify):
7	What's the child's language group?	☐ Bunuba ☐ Goonyandi ☐ Walmajarri/ Wangkatjungka<br>☐ Nykina ☐ Unknown ☐ Other (specify):
8	What's the main language this child speaks at home?	☐ Bunuba ☐ Goonyandi ☐ Walmajarri/ Wangkatjungka<br>☐ Nykina ☐ Unknown ☐ English<br>☐ Kriol ☐ Other (specify):
9	What other languages does this child speak or understand?	☐ Bunuba ☐ Goonyandi ☐ Walmajarri/ Wangkatjungka<br>☐ Nykina ☐ Unknown ☐ Kriol ☐ English<br>☐ Other (specify):<br>**Now go to Q 14**
	**IF NOT ABORIGINAL**	
10	Where were you (the child's mother) born?	☐ Australia ☐ Other (specify):
11	What is your ethnicity/cultural background?<br>(Where did your family originate from?)	☐ Australian ☐ Pacific Islander ☐ Maori ☐ Middle Eastern ☐ Chinese ☐ Japanese ☐ Filipino ☐ Indonesian<br>☐ Other Asian ☐ African ☐ Dutch ☐ English<br>☐ French ☐ German ☐ Italian ☐ New Zealand<br>☐ Scottish ☐ Irish ☐ Indian subcontinent<br>☐ Latin American ☐ Other (specify):
12	Where was this child's (biological) father born?	☐ Australia ☐ Other (specify):
13	What is this child's father's ethnicity/cultural background?	☐ Australian ☐ Pacific Islander ☐ Maori ☐ Middle Eastern ☐ Chinese ☐ Japanese ☐ Filipino ☐ Indonesian<br>☐ Other Asian ☐ African ☐ Dutch ☐ English<br>☐ French ☐ German ☐ Italian ☐ New Zealand<br>☐ Scottish ☐ Irish ☐ Indian subcontinent<br>☐ Latin American ☐ Other (specify):
14	Where does this child live mainly?	☐ Fitzroy Town ☐ Bayulu ☐ Noonkanbah<br>☐ Wangkatjungka ☐ Yakanarra ☐ Unknown<br>☐ Other (specify):
15	Where will this child be mainly living in the dry season?	☐ Fitzroy Town ☐ Bayulu ☐ Noonkanbah<br>☐ Wangkatjungka ☐ Yakanarra ☐ Unknown<br>☐ Other (specify):
16	Where will this child be mainly living in the wet season?	☐ Fitzroy Town ☐ Bayulu ☐ Noonkanbah<br>☐ Wangkatjungka ☐ Yakanarra ☐ Unknown<br>☐ Other (specify):

DATE ....

CHILD ID....................

PART 2 – SCHOOLING AND EDUCATION

Now we want to ask about this child's schooling.

17	**What school does (CHILD) attend?**<br>Which school does this child normally go to?	☐ Fitzroy Crossing ☐ Bayulu ☐ Noonkanbah<br>☐ Wangkatjungka ☐ Yakanarra ☐ None<br>☐ Other (specify):
18	**What grade is (CHILD) in?**<br>What grade is this child in at school now?	☐ Not at school ☐ Pre-school ☐ Grade 1<br>☐ Grade 2 ☐ Grade 3<br>☐ Other (specify):
19	**What other school(s) does (CHILD) attend during different seasons?** (tick all that apply)<br>What other school(s) does this child sometimes go to (this year)? (Tick all the other schools they go to)	☐ Fitzroy Crossing ☐ Bayulu ☐ Noonkanbah<br>☐ Wangkatjungka ☐ Yakanarra ☐ None<br>☐ Other (specify):
20	**How many schools has (CHILD) attended since starting school? – not counting pre-school**<br>How many schools has this child gone to since starting school?	☐ 1 ☐ 2 ☐ 3 ☐ 4 ☐ 5 or more
21	**How often does (CHILD) attend school?**<br>How often does this child attend school?	☐ Not at all ☐ Not much (1 day/week)<br>☐ Little bit (2-3 days/week) **Go to next Q**<br>☐ Most days (4-5 days/week) **Go to Q23**
22	**If they attend school less than 4-5 days per week, why does (CHILD) miss school? (tick all that apply)**<br>Why is this child missing school? (maybe just one reason or more than one)	☐ Family/personal reasons ☐ Illness<br>☐ Skipping school ☐ Suspension<br>☐ School expulsion ☐ Cultural reasons<br>☐ Unknown ☐ Other reasons<br>(specify):
23	**Does (CHILD) receive learning or behaviour support at school? Is (CHILD) in a special class?**<br>Does this child have someone like a special education aide working with them in the classroom because they need extra help? If they do, how much of the time do they get extra help?	☐ No ☐ Unknown<br>☐ Yes (specify what help they get):

PART 3 – YOUR PREGNANCIES AND BIRTHS

Now we'd like to ask a bit about the times you have been pregnant. Is that ok?

24	**How many children have you given birth to altogether?**<br>How many babies have you had altogether?	Number:
25	**How many pregnancies have you had?**<br>Did you lose any babies before they were born?<br>If you have, do you know why you lost them?	☐ Don't want to answer<br>☐ Lost ____babies before birth (number)<br>☐ Miscarriage<br>☐ Abortion / termination<br>☐ Unknown<br>☐ Other (specify):
26	**Was (CHILD) a twin or triplet or more?**<br>Was this child a twin or triplet or more?	☐ No ☐ Twin ☐ Triplet<br>☐ Quadruplet ☐ Unknown
27	**How many children were born before (CHILD)?**<br>How many of your children are older than this child?	Number:
28	**How many children were born after (CHILD)?**<br>How many of your children are younger than this child?	Number:

Now we want to ask about when you were pregnant with this child. Is that ok?

29	**Where did you live when you were pregnant with (CHILD)?**<br>Where were you living when you were pregnant with this child? (tick all the places)	☐ Fitzroy Crossing ☐ Bayulu ☐ Noonkanbah<br>☐ Wangkatjungka ☐ Yakanarra ☐ Unknown<br>☐ Other (specify):
30	**Did you have any check ups during the pregnancy?**<br>Did you have any check ups during the pregnancy?	☐ Yes ☐ No ☐ Unknown
31	**If yes, how far along in the pregnancy was the first check up?**<br>If yes, how far along in the pregnancy was the first check up?	☐ First 3 months ☐ Second 3 months<br>☐ Last 3 months ☐ Unknown

ANTENATAL COMPLICATIONS:
Any problems or special things during the pregnancy

32	**Did you have any problems in the pregnancy with (CHILD)?** Did you have any problems in the pregnancy with this child?		☐ Yes	☐ No	☐ Unknown
	**If yes, what were the problems? (specify all that apply**: If yes, what problems did you have? - maybe one or more than one problem	High blood pressure	☐ Yes	☐ No	☐ Unknown
		Diabetes	☐ Yes	☐ No	☐ Unknown
		Premature rupture of membranes (waters broke early)	☐ Yes	☐ No	☐ Unknown
		Infections	☐ Yes	☐ No	☐ Unknown
		Hospital admission	☐ Yes	☐ No	☐ Unknown
		Anaemia (low iron)	☐ Yes	☐ No	☐ Unknown
		Other (Please specify):			

OTHER ANTENATAL MEDICATIONS AND EXPOSURES:
The next questions are about medicines and drugs in the pregnancy with this child

33	**Were you on any medications during the pregnancy with (CHILD)?** Did you take any medicines during the pregnancy with this child?	☐ Yes	☐ No	☐ Unknown
	**Iron supplements** / Iron medicine?	☐ Yes	☐ No	☐ Unknown
	**Folic acid** / Folic acid or folate?	☐ Yes	☐ No	☐ Unknown
	**Phenytoin (Dilantin)** / The medicine for fits called Phenytoin (Dilantin)	☐ Yes	☐ No	☐ Unknown
	**Valproate (Epilim)** / Another medicine for fits called Sodium Valproate (Epilim)	☐ Yes	☐ No	☐ Unknown
	**Any other medication during this pregnancy?** Did you take any other medicines while you were pregnant with this child?	☐ Yes	☐ No	☐ Unknown
	**Please specify medication.** If yes, what medicine?			
34	**Did you smoke during this pregnancy?** Did you smoke while you were pregnant with this child?	☐ Yes	☐ No	☐ Unknown
35	**Did you chew tobacco during this pregnancy?** Did you chew tobacco while you were pregnant with this child?	☐ Yes	☐ No	☐ Unknown
36	**Did you take any of these drugs during the pregnancy with (CHILD)?** Did you take any drugs like the ones below during the pregnancy?			
	Methadone for heroin addiction	☐ Yes	☐ No	☐ Unknown
	Marijuana (Ganja)	☐ Yes	☐ No	☐ Unknown
	Sniffing solvents e.g. petrol, paint thinner, deodorant spray	☐ Yes	☐ No	☐ Unknown
	Heroin	☐ Yes	☐ No	☐ Unknown
	Cocaine	☐ Yes	☐ No	☐ Unknown
	Speed or Ice	☐ Yes	☐ No	☐ Unknown
	Other (specify):			

ANTENATAL ALCOHOL EXPOSURE:
The next questions are about if you drank alcohol/grog before and during the pregnancy with this child, is that ok? Answering these questions might be a bit hard, but it's really important that you're honest about it. It's not about shame or blame, but about helping children who need help to be as good as they can be. If you start to feel upset we can stop.

37	**Did you drink any alcohol in the 3 months BEFORE the pregnancy with (CHILD)?** Did you drink any alcohol/grog in the 3 months BEFORE you were pregnant with this child?	☐ Yes	☐ No	☐ Unknown
38	**Did you drink any alcohol DURING this pregnancy?** Did you drink any alcohol/grog WHILE you were pregnant with this child?	☐ Yes	☐ No	☐ Unknown
39	**If yes, in which trimester(s) was alcohol consumed (tick all that apply)** If you did drink, how far along in the pregnancy were you when you were drinking? Did you drink in the first three months, the second three months, the last three months, or all the way through?	☐ First 3 months<br>☐ Second 3 months<br>☐ Last 3 months<br>☐ During the entire pregnancy<br>☐ Unknown		
40	**What type of alcohol did you usually drink?** What type of alcohol/grog did you usually drink? Use picture of local drink cans / bottles to specify:	☐ Full strength beer<br>☐ Mid strength beer<br>☐ Light beer<br>☐ Wine	☐ Port/liqueur<br>☐ Spirits<br>☐ Premixed spirits<br>☐ Other (specify):	

 © Fitzpatrick, JP. Elliott, EJ. Peadon, E, Latimer, J. 30.5 2012

41	**Were you drinking cans, bottles, glasses?**<br>What size did you usually drink?<br>Use picture of local drink cups / cans / bottles	☐ Cans<br>☐ Stubbies<br>☐ Long necks<br>☐ Schooner glass<br>☐ Middy glass<br>☐ Wine glass<br>☐ Beer bottles<br>☐ Other (specify):
42	**How many alcoholic drinks did you have on a typical day when drinking?**<br>On the days when you did drink, how many drinks would you have on each day?	☐ 1 - 2 ☐ 3 - 4 ☐ 5 - 6<br>☐ 7 - 9 ☐ 10 or more ☐ Unknown
43	**On average how often did you have a drink containing alcohol?**<br>When pregnant with this child how often did you drink alcohol/grog?	☐ Never<br>☐ Less than once a month<br>☐ Once a month<br>☐ Once every two weeks<br>☐ Once a week<br>☐ 2 or 3 times a week<br>☐ Daily or almost daily
44	**When you were pregnant, how often did you have 6 or more alcoholic drinks on one occasion?**<br>During the whole pregnancy how often did you have 6 or more drinks of alcohol/grog at one time?	☐ Never<br>☐ Less than once a month<br>☐ Once a month<br>☐ Once every 2 weeks<br>☐ Once a week<br>☐ 2 or 3 times a week<br>☐ Daily or almost daily
45	**Will you drink alcohol if you become pregnant again?**<br>If you are pregnant again would you drink alcohol/grog during your pregnancy?	☐ Yes<br>☐ No<br>☐ Don't know / maybe
46	**How sure are you about your answers to these questions about alcohol?**<br>We know it might be hard to remember about grog back before this child was born. Can you say how sure you are about what you've told us?	☐ Very sure<br>☐ Fairly sure<br>☐ Not so sure

PART 4 – THE BIRTH OF THIS CHILD

Now we'd like to ask you about when this child was born.

47	**Where was (CHILD) born?**<br>Where was this child born?	☐ Derby ☐ Fitzroy ☐ Perth ☐ Darwin<br>☐ Other (specify):
48	**How was (CHILD) born?**<br>How was this child born?	☐ Normal vaginal delivery ☐ Vacuum extraction<br>☐ Forceps<br>☐ Elective Caesarean ☐ Emergency Caesarean<br>☐ Other (specify):
49	**How far along in the pregnancy were you when (CHILD) was born?**<br>Was the child born early / at the due date / after the due date?	☐ Early (how early?) __________weeks<br>☐ Late (how late?) __________weeks<br>☐ On time<br>☐ Unknown

BIRTH COMPLICATIONS:
These are some more questions about when the baby was born

50	**How long did (CHILD) spend in hospital after they were born?**<br>How long did this child stay in hospital after they were born?	_____ days ______ weeks ______ months<br>☐ Unknown
51	**Did (CHILD) need any special care (eg phototherapy, antibiotics, admission to special care nursery or newborn intensive care unit?)**<br>Did the baby need any special care after they were born because they were sick?	☐ Yes **If yes, go to next Q**<br>☐ No<br>☐ Unknown **If no or unknown, go to Q 55**
52	**If yes, why was (CHILD) admitted?**<br>What sort of sickness did the baby have?	☐ Breathing difficulties ☐ Feeding difficulties<br>☐ Fits/seizures ☐ Infection<br>☐ Intracranial bleeding ☐ Jaundice<br>☐ Low blood sugar ☐ Withdrawal<br>☐ Unknown ☐ Other (specify):
53	What type or level of special care?	**Level of care:**<br>☐ Level 3 (very sick babies)<br>☐ Level 2 (not quite so sick babies)<br>☐ Special care (not very sick but not as well as normal)<br>☐ Don't know<br>☐ Other (specify):
54	**Which hospital was the baby in for the special care?**	☐ Derby ☐ Fitzroy ☐ Perth ☐ Darwin<br>☐ Other (specify):
55	**Does (did) (CHILD) have any birth defects?**<br>When they were born, did this child have a problem with any of these things:	☐ Yes ☐ No ☐ Unknown<br>**If no or unknown, go to next Q**

	**If yes, what did (CHILD) have?**<br>**Please select all that apply**	Heart defects/<br>Heart	☐ Yes (specify): ____________<br>☐ No ☐ Unknown
		Musculoskeletal (bony) defects/<br>Bone or muscle	☐ Yes (specify): ____________<br>☐ No ☐ Unknown
		Eye defects/<br>Eye	☐ Yes (specify): ____________<br>☐ No ☐ Unknown
		Facial defects/<br>Face	☐ Yes (specify): ____________<br>☐ No ☐ Unknown
		Gastrointestinal (stomach or gut) defects/<br>Guts or stomach	☐ Yes (specify): ____________<br>☐ No ☐ Unknown
		Genital or urinary tract defects/<br>Kidney/ Kidney	☐ Yes (specify): ____________<br>☐ No ☐ Unknown
56	**Other defects**<br>Did this child have any other problems when they were born? If they did, what were they?		☐ Yes (specify): ____________<br>☐ No ☐ Unknown

 © Fitzpatrick, JP. Elliott, EJ. Peadon, E, Latimer, J. 30.5 2012

DATE ....

CHILD ID.....................

PART 5 – GROWTH AND DEVELOPMENT – GROWING UP

The next questions are about the child's development. Development means things like playing sport, writing, talking and making friends. We want to know if you think this child is different from the other children.
We also want to know if this child has been sick from the baby time upward. Is it OK to keep asking questions?

57	**Does (CHILD) have any long-term medical problems requiring ongoing care, including mental health or behavioural problems?**<br>Does this child have any long-term medical problems that need ongoing care, including mental health or behavioural problems?	☐ Yes **If yes, go to next Q**<br>☐ No ☐ Unknown<br>**If no or unknown, go to Q 59**
58	**If yes, specify the problem/s? (all that apply)**<br>What kind of problems does this child have?<br>Please tell us about all the medical problems.<br>(Run through checklist)	Please specify:<br>☐ Cardiovascular (heart)<br>☐ Ear, nose and throat<br>☐ Endocrine (like diabetes or hormones)<br>☐ Gastrointestinal (inside stomach)<br>☐ Neurological (brain, nerves, spine)<br>☐ Respiratory / lung<br>☐ Skin<br>☐ Mental health or behavioural problems<br>☐ Other (specify):
59	**What regular medicines does (CHILD) currently take?**<br>Is this child taking any tablets or medicines for a long time?<br>If yes, what tablets or medicines do they take?	☐ None/no ☐ Unknown<br>☐ Yes (Please list):
60	**Has (CHILD) had any hospital admissions?**<br>Has this child ever been in hospital? (apart from when they were born)	☐ Yes **If yes, go to next Q**<br>☐ No ☐ Unknown<br>**If no or unknown, go to Q 62**
61	**If yes, why was (CHILD) admitted, and which hospital/s?**<br>If yes, why was this child admitted to hospital?<br>Which hospital were they in?	Problem:<br><br>Hospital/s:
62	**Has (CHILD) had a serious head injury?**<br>Has this child ever had a serious head injury?	☐ Yes **If yes, go to next Q**<br>☐ No ☐ Unknown<br>**If no or unknown, go to Q 64**
63	**If yes, what happened to (CHILD)?**<br>If they had a head injury, what happened?	☐ Loss of consciousness (knocked out)<br>☐ Admitted to hospital<br>☐ Skull fracture<br>☐ Bleeding inside the head<br>☐ Other (please specify):
64	**Has (CHILD) ever had have seizures (fits)?**<br>Did this child ever have any fits?	☐ Yes **If yes, go to next Q**<br>☐ No ☐ Unknown<br>**If no or unknown, go to Q 66**
65	**Does (CHILD) have an ongoing seizure disorder?**<br>Does this child have an ongoing problem with fits?	☐ Yes ☐ No ☐ Unknown
66	**Is (CHILD)'s development behind other children their age?**<br>Is this child behind other children in their age group?	☐ Yes **If yes, go to next Q**<br>☐ No ☐ Unknown<br>**If no or unknown, go to Q 68**
67	**If yes, in what areas is (CHILD) delayed? (please tick all that apply)**<br>In what ways is this child behind? (tick all the ones that apply to this child)<br>Are there any other ways you think this child might be behind other children the same age?<br>**(Clarify if child doesn't like doing it, or if they have difficulty)**	☐ Gross motor (big muscles) – running, jumping, throwing<br>☐ Fine motor (small muscles) – drawing, using a pencil, etc<br>☐ Speech and language - speaking and understanding language<br>☐ Literacy – writing or reading<br>☐ Personal/social – making friends<br>☐ Cognitive - thinking and learning<br>☐ Vision / seeing<br>☐ Hearing / hearing<br>☐ Other (please specify):

	**Has the child received therapy or help**	
	**List of referrals needed from the question above**	
**68**	**Has (CHILD) been diagnosed with a Fetal Alcohol Spectrum Disorder (FASD)?**<br>Has anyone told you that this child has FASD?	☐ Yes **If yes, go to next Q**<br>☐ No ☐ Unknown<br>**If no or unknown, go to Q 73**
**69**	**If yes, what is the diagnosis?**<br>What did they tell you this child has?	☐ FAS<br>☐ Partial FAS<br>☐ Alcohol Related Birth Defects<br>☐ Alcohol Related Neurodevelopmental Disorder<br>☐ Unknown<br>☐ Other (specify):
**70**	**Who made the diagnosis?**<br>Who told you this child has FASD?	☐ Hospital doctor ☐ Children's doctor<br>☐ Clinic nurse ☐ Clinic doctor<br>☐ Unknown ☐ Other (specify):
**71**	**Don't ask this question – we will get from records.**<br>**Who else was involved in making the diagnosis?**	Select all that apply<br>☐ Children's doctor ☐ Hospital doctor<br>☐ Clinic nurse ☐ Clinic doctor<br>☐ Occupational Therapist ☐ Physiotherapy<br>☐ Psychologist ☐ Social worker<br>☐ Aboriginal Health Worker<br>☐ Speech Therapy ☐ Unknown<br>☐ Other (specify):
**72**	**Don't ask this question – we will get from records.**<br>**What information was used to make the diagnosis (diagnostic criteria)? Specify all from health record.**	Assessment by:<br>☐ Paediatrician ☐ Occupational Therapist<br>☐ Psychologist ☐ Psychiatrist<br>☐ Speech Therapist ☐ Physiotherapist<br>☐ Other (specify):
**73**	**Is (CHILD) registered with the Disability Services Commission or another disability organisation?**<br>If the child has a disability, are they registered with Disability Services?	☐ Yes: which organisation?<br>____________________<br>☐ No<br>☐ Unknown<br>☐ Other (specify):
**74**	**Does the family receive Carer or Disability Allowance for (CHILD)?** (Carer Allowance is a Centrelink benefit for caring for children with a disability or medical condition)<br>Do you get disability pay for looking after this child?	☐ Yes (specify):<br>☐ No<br>☐ Unknown
**75**	**Do any of your other biological children have a Fetal Alcohol Spectrum Disorder? If so, specify age, gender & diagnosis.**<br>Do any of your other birth children have FASD?<br>If they do, can you tell us their age, gender and the diagnosis?	☐ Yes (specify age, gender and diagnosis of child / children): FAS / FASD / ARND / ARBD<br>____________________<br>☐ Suspected but not diagnosed (specify age and gender of child / children):<br>____________________<br>☐ No ☐ Don't know

PART 6 – ABOUT YOU AND THE FAMILY – BIRTH MOTHER'S DETAILS

76	**What level of school education did you reach?**<br>What was your last year in school?	☐ Primary ☐ Year 10 ☐ Year 11<br>☐ Year 12 ☐ TAFE ☐ Unknown<br>☐ Other (specify):
77	**Post-school study**<br>Have you done any other study after school?	☐ Short courses / certificates<br>☐ TAFE<br>☐ Undergraduate degree<br>☐ Postgraduate degree / diploma<br>☐ No
78	**Are there any medical conditions which run in your family or the birth father's family?**<br>Is there any medical condition that runs in your family or the father's family?	☐ No<br>☐ Unknown<br>☐ Yes (specify):
79	**Do you have any medical problems?**<br>Do you have any medical problem like diabetes or other sickness you see the doctor or nurse for?<br>If you do, what is the problem?	☐ No<br>☐ Unknown<br>☐ Yes (specify):
80	**Do you have any mental health issues?**<br>Do you have any mental health problems like depression or something like that you see the doctor or nurse for?<br>If you do, what sort of problem?	☐ No<br>☐ Unknown<br>☐ Yes (specify):
81	**Do you have any learning problems?**<br>Do you have any problems learning or memorising things?	☐ No<br>☐ Unknown<br>☐ Yes (specify):
82	**Do you currently smoke, drink alcohol or use any drugs?**<br>Do you currently: drink alcohol?<br>smoke cigarettes?<br>use any drugs?	Drink alcohol ☐ Yes ☐ No<br>Smoke cigarettes ☐ Yes ☐ No<br>Other drugs ☐ Yes ☐ No<br><br>☐ No ☐ Don't know
83	**Have you ever had an alcohol related hospital admission?** Have you ever had to be admitted to hospital because of alcohol?	☐ Yes ☐ No ☐ Unknown
84	**Have you ever had an alcohol related injury while intoxicated?**<br>Have you ever been injured while drunk?	☐ No<br>☐ Unknown<br>☐ Yes (specify):
85	**Have you ever had an alcohol related disease? e.g. liver** Have you ever had a disease that the doctor said is because of alcohol - like a liver problem or any other problem inside your body?	☐ Yes ☐ No ☐ Unknown
86	**Have you ever been diagnosed with alcohol dependency requiring treatment?** Have you ever been told by a health worker that you are addicted to alcohol?	☐ Yes ☐ No ☐ Unknown
87	**Do you have a Fetal Alcohol Spectrum Disorder?**<br>Did anyone ever say you had a FASD?	☐ Yes<br>☐ No<br>☐ Unknown<br>☐ Suspected but not diagnosed / maybe
88	**If yes, what is the diagnosis?**<br>If yes, did they say what sort of FASD?	☐ Fetal Alcohol Syndrome<br>☐ Partial FAS<br>☐ ARND<br>☐ Alcohol Related Birth Defects<br>☐ Other<br>☐ Unknown

 © Fitzpatrick, JP. Elliott, EJ. Peadon, E, Latimer, J. 30.5 2012

DATE ....

CHILD ID.....................

PART 7 – AT HOME / BRINGING UP THE CHILD

The next questions are about things in your home that might have affected this child's growing up

89	**How many people (adults and children) usually live with (CHILD)?**<br>How many people (adults and children) usually live in the same house as this child?	Number:
90	**Which adult/s usually lives with (CHILD)? (select all that apply)**<br>Which adults usually live in the same house as this child? (Tick all those that apply)	☐ Birth mother ☐Birth father<br>☐ Foster carers ☐Adoptive family<br>☐ Other (specify)
91	**Unemployment of household members**<br>Do any of those adults have jobs? (tick if any of them do)	☐ Full time ☐ Part time ☐ CDEP<br>☐ Volunteer ☐ No-one works<br>☐ Unknown
92	**Family separation (parents splitting up)**<br>Are this child's father and mother separated from each other?	☐ Yes ☐ No ☐ Unknown
93	**Who has been the adult most involved in growing up this (CHILD)? (please identify relationship to CHILD)**<br>Who has been the main person growing up this child?	☐ Mother ☐ Father ☐ Aunt ☐Uncle<br>☐ Brother ☐ Sister ☐ Grandmother ☐Grandfather<br>☐ Other relative (specify):<br>________________________<br>☐ Other person not a relative (specify relationship to child):<br>________________________<br>☐ Don't know
94	**How many homes has (CHILD) lived in since he/she was born?**<br>How many places has this child lived in since they were born?	Number:
95	**How important have cultural and traditional activities been in (CHILD'S) life?**<br>How important have cultural and traditional activities like hunting, fishing and camping out been in this child's life?	☐ Very important<br>☐ Pretty important<br>☐ Not important<br>☐ Don't know
96	**Is this a good community or neighbourhood for children where they can feel safe?**<br>Is this a good community for children where they feel safe?	☐ Yes ☐ No ☐ Unknown
97	**Are there good places for children to play in this community?**<br>Are there good, safe places for children to go in this community?	☐ Yes ☐ No ☐ Unknown
	These next few questions are about things that could have made the child worry or feel sad while they were growing up. We know these questions might be hard for you. Is it OK to keep going? This is nearly the end of the interview.	
98	**Household worries about money**<br>Are there times when adults in this child's house worry about not having enough money?	☐ Yes ☐ No ☐ Unknown
99	**Not enough food**<br>Are there times when adults in this child's house worry about not having enough food?	☐ Yes ☐ No ☐ Unknown
100	**Overcrowding of home**<br>Do you think there are too many people living in this child's house?	☐ Yes ☐ No ☐ Unknown
101	**Domestic violence**<br>Do adults or parents fight a lot at home?	☐ Yes ☐ No ☐ Unknown
102	**Household member going to jail**<br>Has any family member close to this child been put in prison?	☐ Yes ☐ No ☐ Unknown
103	**Patterns of alcohol and drug taking by adults in the house**<br>We want to ask about alcohol and drug taking by adults in the house	
	**Alcohol or other drug addiction**<br>Is there anyone in the house who has to use alcohol or other drugs every day	☐ Yes ☐ No ☐ Unknown
	Has there been a problem with people bringing grog or drugs in the house so the child felt unsafe or lost sleep all the time?	☐ Yes ☐ No ☐ Unknown
104	**Mental health problems**<br>Is there anyone in the home who may have mental health problems like paranoid or depression or are mental health clients that need counselling?	☐ Yes ☐ No ☐ Unknown
105	**Unexpected death of a household member**<br>Has any family member passed away that have made this child worry or feel sad?	☐ Yes ☐ No ☐ Unknown

DATE ....

CHILD ID......................

106	**Has Department for Child Protection been involved with (CHILD)?** Have DCP or welfare been involved with this child?	☐ Yes	☐ No	☐ Unknown
107	**If yes, is the involvement current or past?** If yes, are DCP involved now or was it before?	☐ Now	☐ Before	☐ Unknown
108	**Has (CHILD) been placed in foster care at any time?** Has welfare at any time taken this child away from you to live somewhere else?	☐ Yes	☐ No	☐ Unknown
109	**If yes, in how many foster homes has this child lived?** How many different families has welfare put this child with?	Number:		

PART 8 – THE LAST FEW QUESTIONS

110	**Where do you go for advice or information about looking after (CHILD)?** If you are worried about growing up your children, where do you go for advice or information?	☐ Media (eg. TV, magazine)<br>☐ Nowhere or self<br>☐ Partner or family member<br>☐ Friends or neighbours<br>☐ Women's resource centre<br>☐ Karrayili<br>☐ Nindilingarri<br>☐ Centrelink<br>☐ Health clinic or hospital<br>☐ School<br>☐ No-one<br>☐ Don't know<br>☐ Other (specify):
111	**Are there any other things you would like to tell me about (CHILD)?** Are there any other things you would like to tell me about this child?	

Thank you so much for being part of this interview. We know some questions have been hard and it has taken a long time. Thank you for giving such personal information.

Nindilingarri, Marninwarntikura and other organisations will work hard to make sure this project helps all children in the Fitzroy Valley.

CONFIDENTIALITY
Remember, the information you give us is confidential between you and the Lililwan Project workers. What you say will be put only on this form without giving it to anyone else outside.
Every person doing these interviews had to sign a confidentiality form saying we are not allowed to tell anyone about what you have told us.
Names on this form will be taken off and replaced with a number so nobody will know whose form it is. Your name or the child's name will not be used, only the number (ID number).

LAST QUESTION

112	Were there any problems with this interview? How do you think we could do it better?	

COMMENTS FROM INTERVIEWER on anything that may be relevant to the accuracy or nature of the data collected from this interview?

Interviewer to fill in at the end of questionnaire:

How reliable was the source of information about antenatal alcohol exposure?
☐ Very reliable – source was the birth mother who recalls her level of consumption well
☐ Very reliable – source is not the birth mother, who directly observed the mother drinking during pregnancy and recalls the level of consumption well
☐ Somewhat reliable – source is the birth mother who does not recall her consumption well
☐ Somewhat reliable – source is not the birth mother and is somewhat sure of the mother's consumption during pregnancy
☐ Alcohol use in pregnancy confirmed from medical records
☐ Not reliable – No reliable history or medical record information available
